# New treatment strategy for chronic low back pain with alpha wave neurofeedback

**DOI:** 10.1038/s41598-022-18931-0

**Published:** 2022-08-25

**Authors:** Keisuke Shimizu, Kazuhide Inage, Mitsuo Morita, Ryota Kuroiwa, Hiroto Chikubu, Tadashi Hasegawa, Natsuko Nozaki-Taguchi, Sumihisa Orita, Yasuhiro Shiga, Yawara Eguchi, Kazuhiko Takabatake, Seiji Ohtori

**Affiliations:** 1 The Future Medicine Education and Research Organization at Chiba University, Chiba University, 1-8-1 Inohana Chuo-ku, Chiba, Chiba 260-8670 Japan; 2 Department of Orthopedic Surgery, Graduate School of Medicine, Chiba University, Chiba, Japan; 3 Division of Rehabilitation Medicine, Chiba University Hospital, Chiba, Japan; 4 Department of Anesthesiology, Graduate School of Medicine, Chiba University, Chiba, Japan; 5 Department of Psychiatry, Graduate School of Medicine, Chiba University, Chiba, Japan; 6 Center for Frontier Medical Engineering, Chiba University, Chiba, Japan; 7 Department of Neurosurgery, Saitama Medical University, Saitama, Japan

**Keywords:** Neuroscience, Psychology, Diseases, Health care

## Abstract

The lifetime prevalence of low back pain is 83%. Since there is a lack of evidence for therapeutic effect by cognitive behavioral therapy (CBT) or physical therapy (PT), it is necessary to develop objective physiological indexes and effective treatments. We conducted a prospective longitudinal study to evaluate the treatment effects of CBT, PT, and neurofeedback training (NFT) during alpha wave NFT. The early-chronic cases within 1 year and late-chronic cases over 1 year after the diagnosis of chronic low back pain were classified into six groups: Controls, CBTs, PTs, NFTs, CBT-NFTs, PT-NFTs. We evaluated the difference in EEG, psychosocial factors, scores of low back pain before/after the intervention. Therapeutic effect was clearly more effective in the early-chronic cases. We found that the intensity of alpha waves increased significantly after therapeutic intervention in the NFT groups, but did not have the main effect of reducing low back pain; the interaction between CBT and NFT reduced low back pain. Factors that enhance therapeutic effect are early intervention, increased alpha waves, and self-efficacy due to parallel implementation of CBT/PT and NFT. A treatment protocol in which alpha wave neurofeedback training is subsidiarily used with CBT or PT should be developed in the future.

## Introduction

While the lifetime prevalence of low back pain in Japan is 83%^1^, and over 75% of cases indicate low back pain with a clear cause according to various examinations, approximately 22% of cases indicate unknown causes as nonspecific low back pain^2^. Since not only organic factors but also psychosocial factors or a lack of exercise, including locomotive syndrome, could affect such nonspecific low back pain, the “Pain Center” has been established in medical institutions throughout Japan. Herein, interdisciplinary treatments with liaisons from various fields such as orthopedic surgery, anesthesiology, psychiatry, and rehabilitation have gained recognition, and comprehensive treatments including cognitive-behavioral therapy (CBT) and physical therapy (PT) are implemented along with standard medication therapy.

It has been reported that both cognitive-behavioral therapy and physical therapy have a certain effect size^3^. Although evidence can be found in support of CBT^4^, the pain-relief effects might be minimal and short lived. Alternatively, a greater effect could be gained for emotional and life disorders^5^, but less so in pain reduction. Because there is a lack of evidence regarding the effectiveness of PT^6–11^, patients may discontinue therapy in many cases as a consequence of unsuccessful therapy. It is necessary to develop a new treatment strategy that is more effective for chronic low back pain.

Therefore, we focused on alpha wave neurofeedback training. The study of neurofeedback on the basis of functional Magnetic Resonance Imaging (fMRI) and EEG has made remarkable progress as an objective evaluation index. Neurofeedback is a method used to visualize brain activity with use of biological signals through fMRI and brain waves, and then control the activity while monitoring function in real time. Among them, a method utilizing alpha brain waves can be widely used in neurofeedback^12^.Previous reports have pointed out the correlation between alpha and beta attenuation and pain intensity^13^. Alpha brain waves neurofeedback may have a clinically meaningful effect on pain intensity in short-term, in recent years, alpha brain waves neurofeedback has been utilized to regulate abnormal brain activity associated with chronic pain^14^.

For these reasons, we conducted an intervention study aimed at examining the difference in efficacy by combining alpha-wave neurofeedback training with treatments usually given to patients with chronic low back pain such as oral administration, CBT, and PT. In addition, since it is important to treat CBT and PT at an early stage^3^, the difference in therapeutic effect will be examined separately for the early stage and the chronic stage.

The treatment of chronic low back pain has been shown to have some effect on CBT and PT, but the evidence is not yet strong. In recent years, the pain treatment effect of alpha wave neurofeedback training has also been reported. Therefore, in this study, we conducted a prospective longitudinal study to evaluate whether the combination of CBT and PT with NFT is more effective than simple treatment and differences between early-chronic cases within 1 year and the late-chronic cases over 1 year after diagnosis of chronic low back pain.

## Methods

### Research participants

Table 1 shows the attributes of the research collaborators. The present study targeted 97 patients with chronic low back pain but no surgical history of low back pain who were referred to the Department of Orthopedics/Anesthesiology and Pain Center at our institution for low back pain after April 2020. Those patients were also recommended to receive CBT and PT since they were diagnosed as no primary organic cause for low back pain on the basis of MRI and neurological symptoms according to four spinal surgeons. The patients were also resistant to standard orthopedic treatments such as medication (i.e., NSAIDs, opioids, gabapentinoids, and anti-depressants) and various block injections. For the registered cases, senior physicians confirmed that MRI findings were not consistent with patient symptoms during a Pain Center conference. The application of CBT and PT was evaluated with the Brief Scale for evaluation of Psychiatric problems in Orthopedic Patients (BS-POP)^15^ and Locomo 25^16^. Standard orthopedic treatments were continued during the intervention trial, but there were no changes in oral medication.

**Table 1:** Demographic characteristics of study participants.

	Controls	CBTs	PTs	NFTs	CBT-NFTs	PT-NFTs
**Age and sex**						
Sex (M/F)	8/12	10/8	5/8	12/8	8/8	4/6
Age (SD)	58.9 (9.81)	57.0 (12.82)	59.9 (12.72)	61.4 (10.12)	63.6 (9.32)	57.8 (11.32)
**Diagnosis**						
None	3	12	4	3	4	1
Mild disc degeneration	4	1	4	2	6	5
Mild idiopathic scoliosis	12	1	5	7	3	5
Mild spondylolisthesis	1	4	0	8	1	0
Others	0	0	0	1	2	0
**Medications (total number)**						
None	0	4	5	4	6	0
NSAIDs	20	10	5	14	7	4
Acetaminophen	10	1	3	9	3	3
Pregabalin/mirogabalin	7	2	0	7	2	3
Tramadol hydrochloride	8	2	0	3	1	2
Duloxetine	8	5	5	5	3	3
**Durations**						
Under 1 years (early-chronic)	7	7	5	13	8	4
Over 1 years (late-chronic)	13	11	8	7	8	6
**Job**						
Employed	4	5	4	12	6	3
Unemployed	16	13	9	8	10	7
**Househeld**						
Alone	13	14	3	12	10	2
With family	7	4	10	8	6	8

The study was conducted in accordance with the Declaration of Helsinki on Ethical Principles for Medical Research involving Human Subjects. The research protocol was approved by the Ethics Committee of Chiba University, and all examinations were conducted in accordance with these guidelines and regulations. We provided a detailed explanation of the study to all patients, and received written informed consent before beginning the study.

### Research design, neurofeedback and EEG analysis

**Figure 1:**
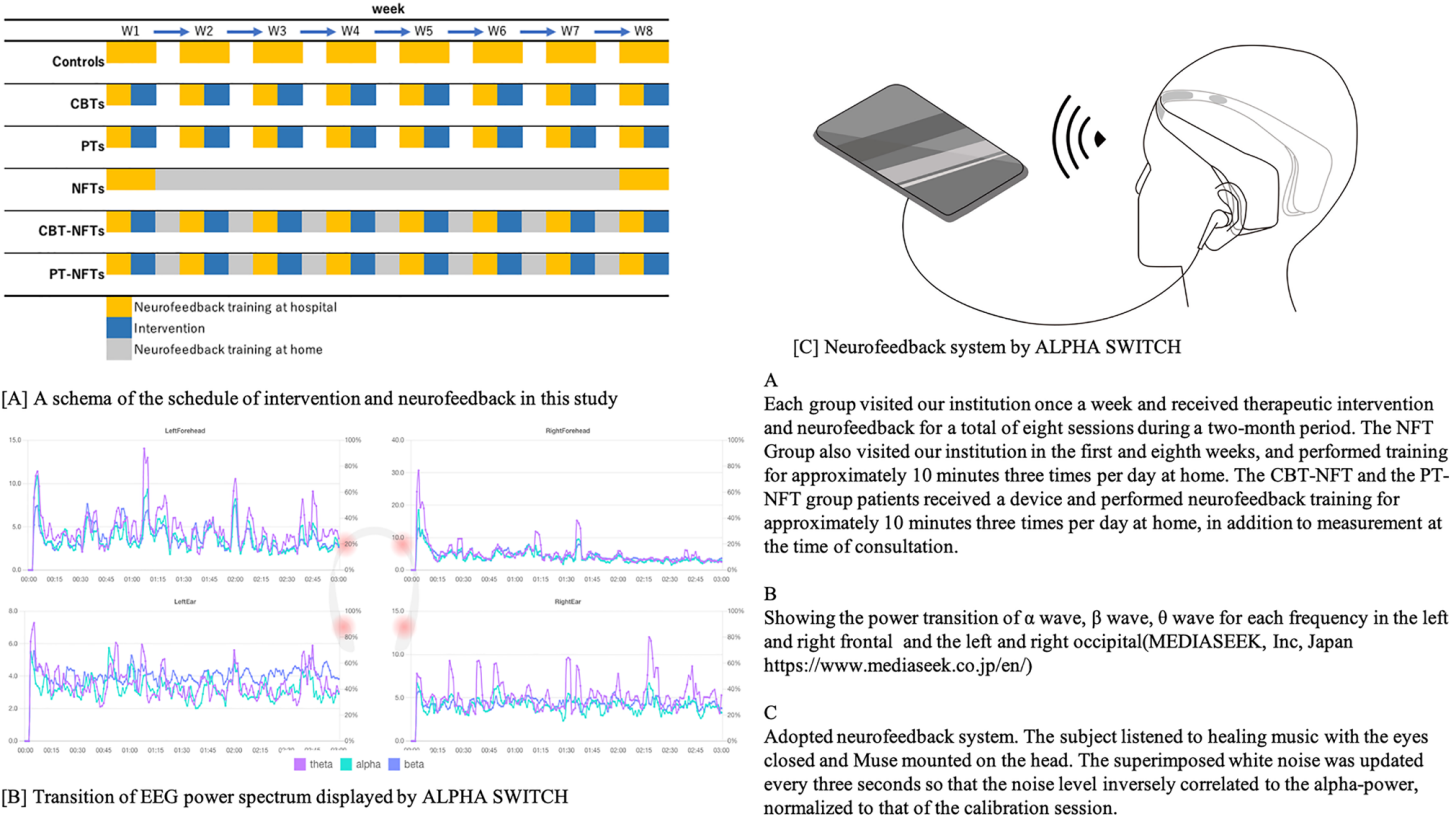
A schema of the schedule of intervention and neurofeedback training in this study and the neurofeedback system.

Figure 1 shows a schema of the schedule of intervention and neurofeedback training in this study and the neurofeedback system. We performed a prospective longitudinal study to evaluate the treatment effects in each group with use of EEG and psychosocial factors as indicators. A researcher who was unaware of the status of each collaborator implemented a completely randomized design through random number calculation with patient IDs. After randomization, our groups comprised 20 cases for the Control Group, 18 for the CBT Group (CBTs), 13 for the Exercise Group (PT), 20 for neurofeedback training (NFTs) group, 16 for the CBT+NFT Group (CBT−NFTs), and 10 cases for the PT+NFT (PT−NFTs) Group.

Each group visited our institution once a week and received therapeutic intervention and neurofeedback for a total of eight sessions during a 2-month period. The NFT Group also visited our institution in the first and eighth weeks, and performed training for approximately 10 min three times per day at home. The CBT−NFT and the PT−NFT group patients received a device and performed neurofeedback training for approximately 10 min three times per day at home, in addition to measurement at the time of consultation.

To perform the measurements and neurofeedback seamlessly without any time lag from the therapeutic intervention, we measured the EEG with a wearable electroencephalograph, then conducted a real-time neurofeedback via a smart-phone application, ALPHA SWITCH ver. 1.3.1 (Mediaseek Inc., Tokyo, Japan, https://www.mediaseek.co.jp/alpha-switch/, available on App Store). Since the application was developed for auditory alpha wave neurofeedback while listening to music and adopted a system to be performed with eyes closed, it is possible to prevent EEG noise contamination caused by eye movement, as is a major problem in EEG measurement.

EEG measurement and neurofeedback were conducted with eyes closed and at resting state in a quiet room. Recording and analysis for EEG and real-time feedback of the analysis results were conducted with the default functions of ALPHASWITCH as follows. Before the feedback session, a 30-s calibration was implemented to measure the baseline. After the voltage data of four electrodes were received from Muse2 256 times/s, the voltage information was stored in the fixed-length circular buffer. We applied DC offset to time-series signals of voltage (768 arrays), and then calculated the inclination of the signal spectrum and the cumulative percentage of noise. To generate power for the alpha frequency band, we performed a Hilbert transformation by applying a bandpass filter from 8 to 13 Hz, then conducted logarithmic transformation for the mean after calculating the magnitude of 768 arrays. For the values after transformation, the outliers were rejected by the Smirnov–Grubbs test. We also calculated the mean and standard deviation for the rejected magnitude values to obtain the magnitude of alpha wave (alpha power) at the time of calibration.

A three-minute feedback session was conducted after the calibration session, and the duration of the feedback session ranged from 3 to 30 min^17–19^. Thus, we conducted the measurement for the shortest time of 3 min to avoid sleep during the feedback session. After processing the signal in the same way as the calibration session, and conducting logarithmic transformation on the magnitude mean of alpha wave, we calculated and set the Z score as normalized Alpha Power (nAP) by utilizing the mean of the magnitude, the mean of the magnitude in the calibration, and the standard deviation after transformation. nAP is an index of magnitude by which alpha can be increased by neurofeedback in comparison with the calibration. We also carried out signal processing and analysis using MATLAB ver. 9.10.0 (The Mathworks, Natick, MA).

Feedback was delivered to each subject auditorily via earphones while listening to healing music, “ Sunbeams”. In feedback session, white noise was superimposed such that the noise level inversely correlated with nAP. Along with the sigmoid function, the volume of white noise was set to zero and the maximum when nAP was 2 and -− 2, respectively. The maximum volume levels of music and white noise were approximately 60–70 and 60 dB, respectively. Participants were instructed to minimize the noise level by increasing alpha-power as much as possible. Participants were instructed to sit down, close their eyes, and meditate, such as creating a relaxing image when the neurofeedback session began.

### EEG device

EEG was measured by Muse2 (InteraXon Inc., Toronto, Canada, https://choosemuse.com/muse-2/), a headband-type wearable EEG device that can be attached to one’s forehead with the ends of the band over both ears. Although the EEG could be easily measured without any special pretreatment on the scalp or forehead, EEG paste was applied for accurate measurement in the present study. Muse2 has four active electrodes and one reference electrode; two active silver chloride electrodes are located on both sides of forehead, and two other active electrodes with conductive silicon rubber are located on both dorsal sides of the auricle to prevent artifacts caused by eye movements, while a reference electrode is located between the two active electrodes on the forehead. Muse2 is based on the international 10–10 system, electrodes are arranged at four locations: TP9, AF7, AF8, and TP10.The sampling rate was fixed at 256 Hz, and the recorded data was immediately transferred to a tablet device (iPad, Apple Inc., San Francisco, CA, USA) via Bluetooth.

### Examination items

We conducted the following examination items for all cases in terms of pain and treatment satisfaction at the time of consultation: Visual Analogue Scale (VAS); the Japanese version of the Oswestry Disability Index (ODI)^20,21^; the Japanese version of the Hospital Anxiety and Depression Scale (HADS)^22,23^; the Japanese version of the Pain Catastrophic Scale (PCS)^24,25^; and the Pain Self-Efficacy Questionnaire (PSEQ)^26,27^.

### Statistical analysis

IBM SPSS Statstics27^®^ (IBM, Armonk, NY) was used to analyze the results. The purpose of this study is to investigate whether the therapeutic effect can be enhanced by combining alpha wave neurofeedback training with CBT and PT according to the previous study. It has also been pointed out that it is important to carry out CBT and PT at an early stage, so statistical analysis is performed on stage (early-chronic/late-chronic) and treatment (Controls, CBTs, PTs, NFTs, CBT-NFTs, PT-NFTs) 2 factors 8 levels ANOVA was performed to examine the low back pain score. And we examined the mean difference in pain and psychological scores before/after therapeutic intervention among the groups. We examined the mean difference in pain and psychological scores before/after therapeutic intervention in each group by conducting a U-test with no presumption of a normal distribution as considering dispersive deflection. Correlation analysis was also performed on the relationship between alpha wave intensity and low back pain scores.

### CBT

The protocol is shown in Table 2. Three clinical psychologists with over 10 years of experience conducted the CBT. The CBT techniques adopted psychoeducation, cognitive reframing, relaxation (abdominal breathing and progressive muscle relaxation), stress management, pacing, and behavioral activation in common use^28,29^. Due to the limited number of reservations, we set a 50-min session/week, for a total of eight sessions.

**Table 2:** Protocol of CBT.

Session	Program	Contents
1	Psychoeducation	Theory of biopsychosocial model
2	Pacing	How to accomplish tasks in a thoughtful and sensible way*
3	Relaxation training	Techniques to decrease stress and muscle tension*
4	Automatic thought	Understand the thought that person has automatically response to pain*
5	Distraction	Distract and draw attention away from pain
6	Cognitive restructuring1	Identify unhelpful thought and increase balanced thinking*
7	Behavioral activation	Increase engagement in rewarding and meaningful activities*
8	Review	Reviewing all CBT program, question and answer session

*Including homework.

### PT

The PT program was a combination of individual PT with a 50-min session/week for a total of eight sessions and daily home-exercises. The contents of the exercise prescription were a multimodal exercise program consisting of lower limb muscle strengthening exercise, motor control training of the trunk, stretching, and aerobic exercise^30–32^. The exercise was set as 40 min/session with the exercise intensity approximately 12–13 on the Borg scale in accordance with the equivalent of METs 4–6^33^.

After an exercise demonstration was provided to the participants in the first session, each participant performed the exercise independently. A physiotherapist assisted the participant to perform his/her exercise with confidence at home every day for 8 weeks, by checking correct techniques with individualized instructions as necessary. For the compliance assessment of the participant’s exercise at home, we used a 5-point Likert scale and the question, “How often did you do the exercises at home?”.

## Results

### VAS and ODI scores for low back pain

The results of VAS scores are shown in Table 3. VAS scores for early-chronic cases indicated significant improvements in CBTs, PTs, CBT-NFTs, and PT-NFTs before/after therapeutic intervention, but there was no significant improvement in Controls and NFTs. We recognized significant improvements for the late-chronic cases in CBT-NFTs and PT-NFTs, but the effect size was clearly higher for the early-chronic cases; in particular, a high effect size was seen in CBT-NFTs (p < 0.01, d = 0.92, 95% CI − 11.97 to − 12.02) and PT-NFTs (p < 0.01, d = 0.68, 95% CI − 15.37 to − 11.04) as the combined use of NFT.

**Table 3:** Average difference in VAS values before/after the each interventions.

	Pre	Post	Amount of change	*p *value	Effect size (d)	95% CI
	*M (SD)*	*M (SD)*				
**Early-chronic**						
Controls	70.7 (15.60)	71.7 (16.30)	1.00	0.60	0.06	
**CBTs**	**72.2 (15.40)**	**66.3 (14.21)**	**− 5.89**	0.04**	**0.33**	**− 7.36 to − 4.41**
**PTs**	**66.3 (14.21)**	**56.5 (14.74)**	**− 9.83**	0.00**	**0.47**	**− 12.81 to − 6.84**
NFTs	68.9 (15.71)	65.2 (17.48)	**− **3.72	0.32	0.22	
**CBT-NFTs**	**69.6 (14.65)**	**53.6 (19.73)**	**− 16.00**	0.00**	**0.92**	**− 19.97 to − 12.02**
**PT-NFTs**	**72.2 (18.54)**	**58.8 (20.89)**	**− 13.39**	0.00**	**0.68**	**− 15.37 to − 11.04**
**Late-chronic**						
Controls	72.4 (15.02)	73.6 (6.82)	1.17	0.67	0.10	
CBTs	74.6 (14.35)	69.9 (20.34)	**− **4.67	0.13	0.27	
PTs	69.0 (12.78)	65.2 (14.10)	**− **3.84	0.21	0.29	
NFTs	68.6 (15.14)	69.2 (13.05)	**− **0.61	0.87	0.04	
**CBT-NFTs**	**73.8 (7.30)**	**66.8 (12.58)**	**− 7.00**	0.00**	**0.68**	**− 8.39 to − 5.60**
**PT-NFTs**	**75.0 (15.80)**	**68.1 (15.66)**	**− 6.89**	0.04*	**0.44**	**− 8.88 to − 4.26**

Significant values are in [bold].

*p < 0.05.

**p < 0.01.

Results of two-way ANOVA with 2 factors and 8 levels consisting of treatment stage (early/late-chronic) and intervention group (control, CBTs, PTs, NFTs, CBT-NFTs, PT-NFTs), main effect were found in treatment stage (*F* [1,96] = 8.59, *p* < 0.01,η^2^ = 0.11) and the intervention group (*F* [5,96] = 6.84, *p* < 0.05, η^2^ = 0.09) respectively. And the interaction between these terms was significant(*F* [5,85] = 7.24, *p* < 0.01, η^2^ = 0.12). According to multiple comparison (Tukey), CBTs, PTs, CBT-NFTs, and PT-NFTs showed a significant difference between the early-chronic and late-chronic (*p* < 0.01) cases, and it was found that the VAS score was lower in the early-chronic cases.

ODI scores are shown in Table 4, and indicated a similar trend to the VAS scores. We recognized a high effect size in CBT-NFTs (*p* < 0.01, d = 1.21, 95% CI − 13.81 to − 9.84) and PT-NFTs (*p* < 0.01, d = 0.80, 95% CI − 8.22 to − 6.31) for the early-chronic cases. Result of a two-way ANOVA similar to the above, a main effect was found in the treatment stage (*F* [1,96] = 8.26, *p* < 0.01, η^2^ = 0.10) and the intervention group (*F* [5,96] = 6.93, *p* < 0.01, η^2^ = 0.07). And the interaction between these terms was significant(*F* [5,85] = 9.24, *p* < 0.01, η^2^ = 0.11). According to a multiple comparison (Tukey), PTs and CBT-NFTs showed a significant difference between the early-chronic cases and the late-chronic cases (*p* < 0.01), and it was found that the pain ODI score was lower in the early-chronic cases.

**Table 4:** Average difference in ODI values before/after the each interventions.

	Pre	Post	Amount of change	*p *value	Effect size (d)	95% CI
	*M (SD)*	*M (SD)*				
**Early-chronic**						
Controls	38.39(7.63)	39.28(6.80)	0.39	0.47	0.19	
**CBTs**	**38.8 (9.66)**	**32.7 (12.95)**	**− 6.11**	0.04*	**0.54**	**− 7.10 to − 5.11**
**PTs**	**34.8 (10.70)**	**29.0 (12.14)**	**− 5.89**	0.00**	**0.52**	**− 7.12 to − 4.65**
NFTs	33.3 (9.03)	33.6 (9.57)	0.28	0.82	0.03	
**CBT-NFTs**	**39.8 (8.44)**	**28.0 (11.11)**	**− 11.83**	0.00**	**1.21**	**− 13.81 to − 9.84**
**PT-NFTs**	**34.4 (10.20)**	**27.1 (8.75)**	**− 7.27**	0.00**	**0.80**	**− 8.22 to − 6.31**
**Late-chronic**						
Controls	40.6 (8.78)	42.4 (7.07)	1.83	0.61	0.23	
**CBTs**	**37.8 (9.82)**	**34.1 (10.16)**	**− 3.78**	0.04**	**0.33**	**− 4.54 to − 3.01**
**PTs**	**36.9 (9.70)**	**33.0 (7.98)**	**− 3.94**	0.02**	**0.44**	**− 4.75 to − 3.12**
NFTs	38.2 (7.09)	36.1 (7.86)	**− **2.17	0.27	0.28	
**CBT-NFTs**	**41.9 (8.25)**	**38.7 (7.18)**	**− 3.22**	0.02*	**0.68**	**− 4.21 to − 2.22**
**PT-NFTs**	**35.1 (9.22)**	**29.8 (7.61)**	**− 5.22**	0.00**	**0.62**	**− 6.81 to − 3.11**

Significant values are in [bold].

*p < 0.05.

**p < 0.01.

### Mean comparison of nAP with neurofeedback training

**Figure 2:**
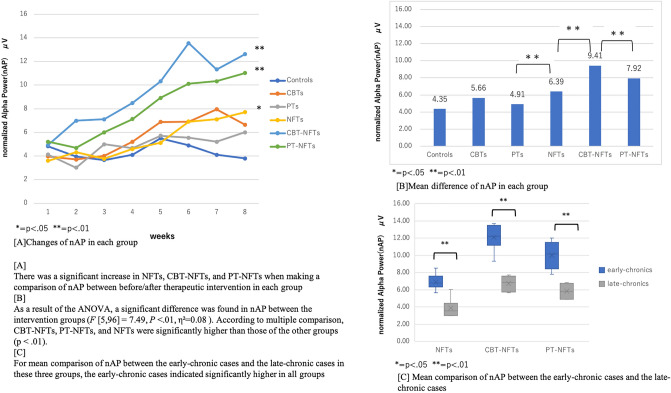
Mean comparison of nAP with neurofeedback training.

The results are shown in Fig. 2. We found that there was a significant increase in NFTs, CBT-NFTs, and PT-NFTs when making a comparison of nAP between before/after therapeutic intervention in each group (Fig. 2A). As a result of the one-way ANOVA(Fig. 2B), a significant difference was found in nAP between the intervention groups (*F* [5,96] = 7.49, *p* < 0.01, η^2^ = 0.08). According to multiple comparison, CBT-NFTs, PT-NFTs, and NFTs were significantly higher than those of the other groups (p < 0.01). For mean comparison of nAP between the early-chronic cases and the late-chronic cases in these three groups, the early-chronic cases indicated significantly higher in all groups (Fig. 2C).

Table 5 shows the relationship between mean of nAP and pain. In Early-chronic cases, CBT-NFTs showed a significantly high negative correlation with VAS (r = 0.71, p < 0.05) and ODI (r = 0.72, p < 0.01). It can be said that the higher the nAP, the lower the back pain score. Both VAS and ODI showed moderate negative correlations for PT-NFTs. On the other hand, in NFTs that showed high values in nAP, no correlation was observed for both VAS and ODI, and it could not be said that low back pain was alleviated if nAP was high. Parallel interventions of CBT and NFT at an early stage to reduce low back pain are required. However, in late-chronic cases, there was no high correlation between nAP and back pain. It can be said that increasing nAP at an early stage is related to reducing chronic low back pain.

**Table 5:** Correlation coefficient about mean of nAP and VAS/ODI score in each group.

	VAS		ODI			VAS		ODI	
	r	*p*	r	*p*		r	*p*	r	*p*
	**Early-chronic**				**Late-chronic**				
Controls	− 0.22	0.13	− 0.12	0.42	Controls	− 0.31	0.21	− 0.39	0.61
CBTs	− 0.41	0.11	− **0.51**	**0.04**	CBTs	− 0.34	0.41	− 0.41	0.12
PTs	− 0.36	0.21	− 0.33	0.25	PTs	− **0.41**	**0.04**	− 0.41	0.11
NFTs	− 0.11	0.32	− 0.19	0.12	NFTs	− 0.25	0.32	− 0.28	0.31
CBT-NFTs	− **0.71**	**0.02**	− **0.72**	**0.01**	CBT-NFTs	− **0.61**	**0.01**	− 0.58	0.06
PT -NFTs	− **0.54**	**0.04**	− **0.42**	**0.02**	PT-NFTs	− 0.44	0.07	− 0.36	0.09

Significant values are in [bold].

### Psychological factors

The results are shown in Table 6. After the examination of mean difference for the scores before/after therapeutic intervention in each Group, PCS, PSEQ, and HADS (depression) showed a significant improvement in CBTs, PTs, and CBT-NFTs, while PCS and PSEQ showed a significant improvement in PT-NFTs for the early-chronic cases. Yet, PCS, PSEQ, HADS showed a significant improvement in CBT-NFTs, but only PSEQ showed a significant improvement in CBTs, PTs and PT-NFTs for the late-chronic cases.

**Table 6:** Changes in psychological score before/after intervention.

Psychological scales	Early-chronic				Late-chronic			
	Pre	Post	*p*	*R *with* nAP*	Pre	Post	*p*	*R *with* nAP*
**Controls**								
PCS	41.4 (± 5.91)	40.0 (± 4.66)	0.70	− 0.11	46.1 (± 8.31)	44.4 (± 8.10)	0.69	− 0.13
PSEQ	21.9 (± 6.01)	19.6 (± 7.73)	0.69	0.13	16.7 (± 7.62)	15.4 (± 7.95)	0.72	0.16
HADS (depression)	8.6 (± 1.87)	9.0 (± 2.30)	0.71	− 0.02	10.2 (± 2.00)	10.0 (± 2.65)	0.81	− 0.03
HADS (anxiety)	9.3 (± 2.11)	10.0 (± 3.74)	0.67	− 0.09	11.3 (± 3.68)	10.2 (± 3.11)	0.32	− 0.12
**CBTs**								
PCS	**38.0 (± 7.06)**	**28.3 (± 8.89)**	0.00**	− 0.71	42.6 (± 9.31)	39.0 (± 9.80)	0.06	− 0.49
PSEQ	**16.7 (± 4.33)**	**25.7 (± 8.91)**	0.00**	0.87	**13.9 (± 6.96)**	**18.3 (± 4.77)**	0.04*	0.84
HADS (depression)	**10.0 (± 2.37)**	**6.4 (± 1.93)**	0.04*	− 0.89	9.7 (± 3.12)	9.0 (± 1.22)	0.34	− 0.59
HADS (anxiety)	8.8 (± 1.55)	8.0 (± 1.42)	0.71	− 0.19	9.0 (± 2.76)	8.5 (± 1.99)	0.67	− 0.18
**Exercises**								
PCS	**41.9 (± 6.66)**	**30.2 (± 6.78)**	0.04*	− 0.90	42.5 (± 10.27)	36.3 (± 5.48)	0.05	− 0.88
PSEQ	**24.0 (± 7.29)**	**31.1 (± 6.73)**	0.03*	0.88	**20.0 (± 8.11)**	**25.6 (± 4.88)**	0.04*	0.82
HADS (depression)	**7.9 (± 2.17)**	**5.4 (± 2.03)**	0.04*	− 0.69	11.2 (± 2.03)	10.3 (± 1.09)	0.32	− 0.32
HADS (anxiety)	9.9 (± 1.98)	9.0 (± 2.02)	0.46	− 0.01	10.0 (± 3.16)	9.9 (± 2.53)	0.72	− 0.10
**NFTs**								
PCS	36.0 (± 5.17)	39.1 (± 6.33)	0.54	− 0.15	39.9 (± 7.77)	39.1 (± 4.10)	0.80	− 0.08
PSEQ	22.7 (± 5.41)	23.1 (± 3.30)	0.66	0.03	16.7 (± 4.50)	16.1 (± 4.11)	0.81	0.02
HADS (depression)	10.3 (± 1.99)	10.2 (± 2.88)	0.71	− 0.10	13.9 (± 3.41)	12.2 (± 3.02)	0.76	− 0.11
HADS (anxiety)	9.1 (± 1.32)	7.0 (± 1.93)	0.19	− 0.19	11.2 (± 4.55)	11.1 (± 2.99)	0.84	− 0.02
**CBT-NFTs**								
PCS	**43.0 (± 5.16)**	**29.2 (± 6.79)**	0.00**	− 0.91	**45.1 (± 7.21)**	**35.1 (± 8.12)**	0.04*	− 0.87
PSEQ	**22.1 (± 8.18)**	**30.2 (± 5.72)**	0.00**	0.82	**17.6 (± 9.20)**	**26.4 (± 4.14)**	0.03*	0.84
HADS (depression)	**8.9 (± 3.22)**	**4.0 (± 1.92)**	0.04*	− 0.78	**13.9 (± 5.20)**	**7.2 (± 3.18)**	0.05*	− 0.81
HADS (anxiety)	8.5 (± 2.12)	8.5 (± 1.95)	0.16	− 0.01	16.3 (± 6.62)	10.2 (± 3.27)	0.09	− 0.73
**PT-NFTs**								
PCS	**43.1 (± 8.02)**	**32.9 (± 7.77)**	0.05*	− 0.72	44.2 (± 10.21)	36.3 (± 10.27)	0.11	− 0.54
PSEQ	**18.9 (± 8.55)**	**33.9 (± 5.31)**	0.00**	0.93	**16.5 (± 7.52)**	**25.2 (± 7.22)**	0.04*	0.65
HADS (depression)	8.4 (± 2.03)	7.0 (± 1.64)	0.12	− 0.14	13.9 (± 6.23)	10.2 (± 3.58)	0.23	− 0.56
HADS (anxiety)	9.1 (± 1.77)	9.0 (± 2.07)	0.56	− 0.02	14.5 (± 4.31)	14.3 (± 4.01)	0.61	− 0.49

Significant values are in [bold].

*p < 0.05.

**p < 0.01.

Table 6 shows the correlation between nAP and psychological scores. PSEQ showed a high correlation in early-chronic cases of CBTs, PTs, CBT-NFTs, and PT-NFTs. The higher the PSEQ score, the higher the nAP. In late-chronic cases, CBT-NFTs and PT-NFTs showed a moderately significant correlation with PSEQ, but no psychological factors showed a high correlation.

## Discussion

The treatment of low back pain was clearly more effective in the early-chronic cases compared to late-chronic cases. The intensity of alpha waves increased significantly after therapeutic intervention in the NFT groups, but did not reduce low back pain. However, the interaction between CBT and NFT reduced low back pain. PSEQ indicated a high correlation with alpha waves.

Since we found that there was a clear difference in therapeutic effect between early-chronic cases within 1 year and late-chronic cases over 1 year after diagnosis, it can be said that both CBT and PT could be effective in reducing pain during the early stage. nAP is significantly higher in the CBT-NFTs and PT-NFTs in early-chronic cases than in other groups, and it can be said that the intensity of alpha waves can be increased by using CBT and PT in combination with NFT at an early stage. In particular, the CBT-NFTs show a high correlation with nAP and VAS/ODI, and can be said to be extremely effective as a treatment. However, even if the alpha wave increases, it cannot be said that the back pain is improved by itself, and we should use a combination of CBT or PT at an early stage.

The tendency is the same for psychosocial factors. In early-chronic cases, CBT and PT improve many psychological factors, but become difficult to improve more than 1 year after chronicity. The correlation between psychosocial factors and nAP is particularly high in PSEQ, and it is considered that the increase in self-efficacy in early-chronic cases affects the increase in alpha waves. Although NFT itself did not have a low back pain-reducing effect, the effect size was particularly high when NFT and CBT were performed in parallel, and it can be said that NFT plays a role in enhancing the effect of CBT.

The combined treatment of NFT and CBT or PT was more effective than regular CBT or PT, probably because of increased pain self-efficacy (PSEQ). Pain self-efficacy is an indicator of the strength of self-confidence that you can control pain yourself. In addition, NFT is not a treatment that is passively received, but a proactive treatment that actively controls the state of the brain. Therefore, it is highly possible that the relaxation and mindfulness effects of NFTs have cultivated a sense of self-control of pain. In addition, CBT and PT are usually treatments that are performed at the time of visit and cannot be performed at home. The fact that NFTs can be performed at home may be one of the factors that have increased the therapeutic effect. In the past, CBT has often been applied as a last resort when all other treatments have failed. Yet, the present study demonstrated that low back pain should be treated from the early stage with therapeutic intervention as being similar to exercise therapy due to the obvious reduction in treatment effects 1 year or longer after chronicity. In the present study, we also found that the neurofeedback training could play a role in improving the effectiveness of existing treatments. Thus, it is important to implement a protocol to enhance pain self-efficacy by CBT and exercise therapy intervention while monitoring EEG with NFT during the early stage after chronicity. A number of the past studies for chronic low back pain ignore the duration of chronic low back pain and include it as a single category. However, since we recognized that therapeutic response could be completely different in chronic low back pain between the early sage and the prolonged condition after chronicity, it is expected that the results might lead to the establishment of therapeutic application criteria and the development of new treatments with the consideration of patient’s detailed psychosocial factors by disease duration.

## Limitations

For the limitations in the present study, we only selected patients through the background of psychosocial factors including patients with extreme back pain but no issues in detailed imaging and neurological examination. Yet, we continuously need to consider how therapeutic effects and the strength of alpha waves would affect the health of patients who receive no influence of psychosocial factors or lesser intensity of low back pain. Furthermore, the reason why the combination of NFT and CBT or PT was effective could be explained psychologically from the correlation with PSEQ, but the neurophysiological mechanism has not been clarified. In the future, it is necessary to continue research by adding fMRI and SPECT.

## Conclusion

It is certainly important that patients with chronic low back pain receive CBT and PT during the early stage, and we recommend avoiding follow-up with oral medication due to a possible major cause of intractable conditions. Since enhancement in alpha waves and self-efficacy during the early stage can become a factor for increasing therapeutic effectiveness, it is expected that therapeutic effect can be increased by developing a treatment protocol with a subsequent use of alpha wave neurofeedback training for enhancing self-efficacy in the future. We need to conduct further research for therapeutic effect and the strength of alpha waves for patients with no influence of psychosocial factors as well as patients with a lower intensity of low back pain.

## Data Availability

The datasets during and/or analyzed during the current study available from the corresponding author on reasonable request.
